# Educational and Clinical Applications of a Web- and Android-Based Telemedicine Platform to Expand Rural Health Care in Ecuador

**DOI:** 10.1089/tmr.2024.0091

**Published:** 2025-03-05

**Authors:** Leonel Vasquez-Cevallos, Andrew Mitchell, Susana Muñoz-Hernández, Ángel Herranz-Nieva, Ana Garcia-Mingo, Paula De Corral-San Martin, Mijail Castro, Paul E.D. Soto-Rodriguez, Franklin Parrales-Bravo, Rosangela Caicedo-Quiroz

**Affiliations:** ^1^ SIMUEES Simulation Clinic, Universidad Espíritu Santo (UEES), Samborondón, Ecuador.; ^2^ ETSI Informáticos, Universidad Politécnica de Madrid, Madrid, Spain.; ^3^ Asociación Amigos del Cayapas-Cayapa Pichulla Kumani, Madrid, Spain.; ^4^ Whittington Health NHS Trust, London, UK.; ^5^ Departamento de Física, Instituto de Estudios Avanzados IUDEA, Universidad de La Laguna, Tenerife, Spain.; ^6^ Universidad Bolivariana del Ecuador, Durán, Ecuador.

**Keywords:** telemedicine, teleconsultation, rural health care, Web- and Android-based Telemedicine Platform, clinical validation, medical education validation

## Abstract

**Introduction:**

The Web- and Android-based Telemedicine Platform (WATP) is a digital tool designed to facilitate remote medical consultation and data exchange through mobile devices. It addresses health care gaps in underserved rural regions, such as Ecuador, where access to specialized care is limited. This study validated the platform in the Ecuadorian context, focusing on its use in rural clinical settings and its potential integration into academic and health care institutions as a scalable solution for nationwide implementation.

**Materials and Methods:**

A mixed-methods approach was used, including technical, clinical, and educational validation. Technical validation involved 10 general practitioners and five specialists who evaluated task completion times, error rates, and user satisfaction. Clinical validation analyzed three teleconsultations, one pediatric and two dermatological, conducted between October 2022 and December 2023, with a focus on diagnostic precision and case clarity. The educational validation involved 17 final-year medical students, 2 faculty members, and 2 observers in a gynecology course in a simulation center to evaluate its impact on learning outcomes.

**Results:**

Technical validation demonstrated low error rates, high user satisfaction, and average task completion times of 5 min for general practitioners and 3 min for specialists. Clinical validation achieved 100% diagnostic accuracy through cross-validation with five independent specialists. Educational validation showed significant improvements in the students’ diagnostic skills and clinical case documentation abilities.

**Conclusion:**

This study highlights the potential of WATP to improve health care access and enhance diagnostic skills among medical students, offering a scalable solution tailored to rural challenges in Ecuador.

## Introduction

The provision of quality health care in rural regions poses a global challenge, as these underserved areas frequently lack specialized services and trained professionals.^1–3^ In Ecuador, such disparities are particularly pronounced, with rural populations encountering substantial barriers to accessing adequate health care.^3,4^ Mobile telemedicine platforms offer scalable solutions, enabling teleconsultations between general practitioners and specialists to improve health care access.^5–7^ However, challenges persist, including inadequate infrastructure, regulatory obstacles, cultural resistance, and workforce shortages.^8–10^ In Ecuador, additional barriers like limited internet, unreliable electricity, and low telemedicine awareness further hinder adoption.^11^

Previous efforts to address these barriers included a study conducted from 2012 to 2016 in Ecuador, which validated a web-based telemedicine platform (TMP) as an effective tool for clinical case discussions and medical training.^12,13^ However, this solution was limited to web-based interactions, leaving mobile-based platforms unexplored, despite their potential for broader applications. Mobile systems can complement TMP by offering functions such as appointment scheduling, personalized care plans, and offline usability.^14^ Integrating these capabilities could provide a more comprehensive telemedicine solution for underserved areas.^8^

Inspired by these developments, the Amigos del Cayapas Medical Non-Governmental Organization (NGO)—Amigos del Cayapas-Cayapa Pichulla Kumani (AAC-CPK), located in Spain, has worked since 2012 to improve access to health care for the rural Chachi Indigenous communities in Esmeraldas, Ecuador.^15^ In 2020, a telemedicine project was launched along the Cayapas River in response to a 2019 hygiene study that identified key diseases such as dermatitis, parasitosis, and respiratory infections. The initial telemedicine platform, hosted on Amazon Web Services, saw limited adoption due to its lack of features and user engagement.^16^ In 2022, a new Web- and Android-based Telemedicine Platform (WATP) was introduced to overcome these limitations, offering offline functionality to meet the needs of rural physicians in areas without internet connectivity.^17^

This study, in collaboration with the AAC-CPK, presents a WATP designed to improve health care access for underserved rural communities in Ecuador. The platform includes offline capabilities and an educational element targeted at final-year medical students, and it was validated through clinical and educational assessments. The iterative design integrates input from health care providers to address the requirements of settings with limited resources. The Amigos del Cayapas telemedicine initiative seeks to extend its reach to rural areas, improving its capabilities for wider medical usage and collaboration across other disciplines.

## Materials and Methods

### The web- and android-based telemedicine platform

The telemedicine platform was developed collaboratively by research teams from the Universidad Politécnica de Madrid, the Instituto de Estudios Avanzados IUDEA, and the SIMUEES Simulation Clinic, with significant contributions from the NGO AAC-CPK. The NGO’s medical members played a key role in designing the platform and funded field visits to evaluate its performance in rural areas.

Telemedicine platform was designed using iterative and user-centered methodologies to address health care challenges in rural Ecuador. The platform includes an Android-Based Telemedicine app (ABTapp) for general practitioners and a web platform for specialists, designed to support asynchronous teleconsultation. ABTapp’s offline functionality addresses the connectivity challenges in rural areas, while the web platform enables specialists to efficiently review and manage cases in settings with stable internet access. This approach ensures that both user groups have tools tailored to their specific needs.^18,19^

#### System architecture

**FIG. 1.**
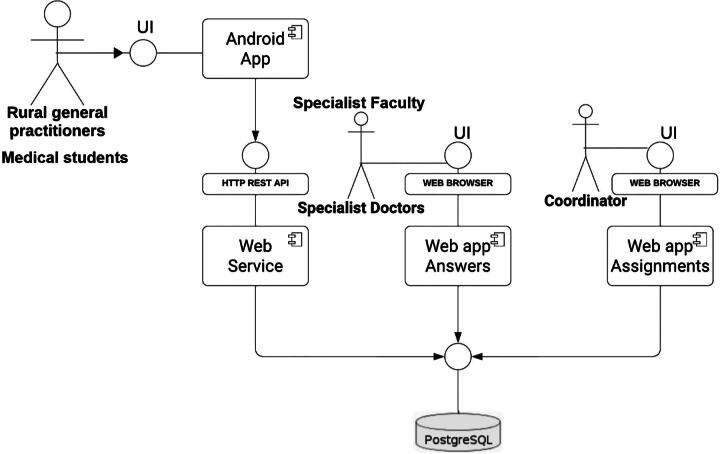
UML diagram of the system architecture and UI. UI, user interface; UML, unified modeling language.

The platform has undergone continuous refinement through agile development, with key updates provided by user feedback to improve usability, scalability, and reliability. The UML diagram of the system shows its architecture and user interface, offering a clear framework for database interactions and workflows (Fig. 1).^20^

#### Development process

The development process followed an iterative approach that included continuous feedback from end users to refine the system.^21,22^ It began with requirement analysis, where interviews and surveys with rural and specialist physicians identified specific health care challenges.^7,8,12^ Design focused on creating wireframes and mockups to visualize the system layout and improve user interactions.^23^ The system was developed using agile methodologies, allowing iterative improvements through regular sprint reviews and user feedback.^24^ Finally, comprehensive testing, including unit, integration, and user acceptance testing, ensured functionality and usability, enhancing system reliability, and user satisfaction.^25^

#### Implementation and testing design

The telemedicine platform utilizes robust and scalable technologies optimized for rural health care needs. The mobile application was developed using Android Studio with Kotlin and ObjectBox, enabling offline data access and efficient storage through a NoSQL database. Communication with the central server was facilitated via a RESTful API to ensure reliable data transmission. The web platform leverages the Elixir programming language on the Phoenix framework, which is known for its scalability and fault tolerance, alongside PostgreSQL for managing large data volumes. To protect sensitive clinical data, security measures include HTTPS for secure transmission, JSON Web Tokens for authentication, and encryption for data storage, adhering to health informatics standards.^26–29^

**FIG. 2.**
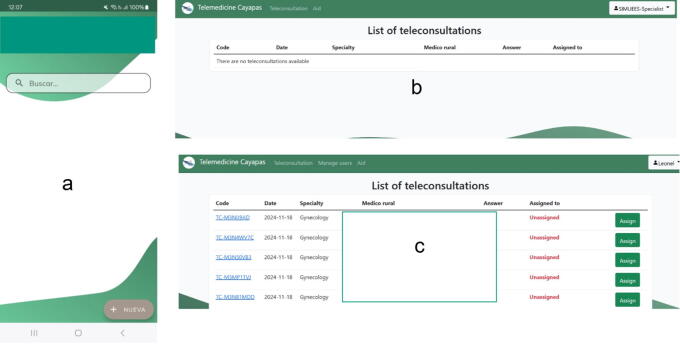
User interfaces of the telemedicine platform: ABTapp dashboard **(a)**, faculty web dashboard **(b)**, and coordinator web dashboard **(c)**. ABTapp, Android-Based Telemedicine app.

Rural general practitioners and advanced medical students access the platform via the ABTapp or web version, allowing flexibility to upload clinical data and perform teleconsultations. Specialists, faculty, and coordinators exclusively use the web platform for reviewing cases, providing feedback, and managing consultations. The ABTapp is available on Google Play.^30^ Access to the web platform is managed through the TEDECO Cayapas login portal, a cooperation and development group from the Universidad Politécnica de Madrid specializing in information and communication technologies.^31^ User registration and coordinator validation ensure secure access and functionality. The user interfaces for general practitioners and specialists are showcased, emphasizing user-centered design and system functionality (Fig. 2).

### Evaluation methodology

This study employed a mixed-methods approach to validate the platform in technical, clinical, and educational contexts.^32,33^

#### Study design and participants

Technical validation was conducted with 10 general practitioners and 5 specialists to assess task completion times, error rates, and user satisfaction in simulated conditions. Clinical validation included three teleconsultations—one pediatric and two dermatological—conducted between October 2022 and December 2023. Five independent specialists validated the cases to ensure diagnostic accuracy and clarity. Educational validation included 17 final-year medical students who completed teleconsultations during a gynecology course. Two faculty members evaluated the accuracy and quality of the submissions.

Participants were selected through voluntary collaboration and all provided informed consent. Both rural general practitioners and specialists from the NGO AAC-CPK participated in the clinical validation. For the educational validation, faculty experienced in the medical simulation were selected, particularly those using SIMUEES in gynecology courses, which frequently utilize the simulation center.

#### Teleconsultation protocol

**FIG. 3.**
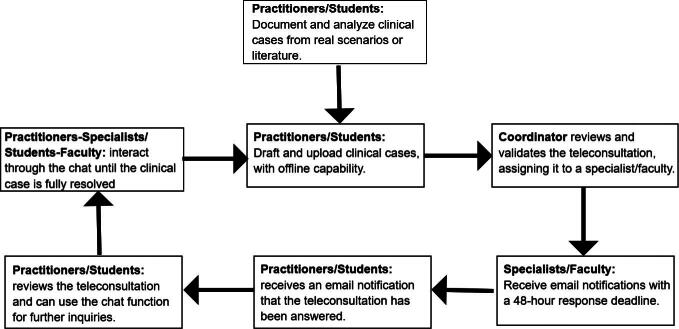
Workflow for specialists/faculty and general practitioners/students during clinical teleconsultation submissions.

The protocol begins with rural general practitioners or students drafting clinical cases, which are submitted via the ABTapp or web platform. Each submission included the required fields covering thematic blocks, such as consultation details, medical history, and diagnostic information. The workflow is designed to ensure consistency, traceability, and efficiency through the following steps: (1) Initial review: A coordinator verifies the completeness and quality of the case before assigning it to a specialist. (2) Assignment and review: Specialists evaluate the case, request additional information via instant messaging if needed, and provide a diagnosis or recommendation. (3) Response and follow-up: The coordinator ensures that responses are delivered within 48 h and reassigns cases when necessary. (4) Bidirectional communication: General practitioners or students can ask follow-up questions and confirm responses, fostering effective collaboration. (5) System integration: The platform incorporates essential features, such as built-in chat functionality and secure access through an authenticated portal. This structured workflow supports the traceability, quality, and efficiency of the teleconsultation processes. The interaction steps for specialists, faculty, general practitioners, and students during the submission and resolution of teleconsultations are outlined (Fig. 3).

For educational validation, advanced medical students completed four teleconsultations during their academic term, simulating diagnostic scenarios to develop competencies. Cases were based on literature or hospital practice in the gynecology course. Faculty evaluated submissions as part of course grading, focusing on timely delivery and case quality. The platform’s adaptability for health care delivery and medical education is demonstrated through the workflow for specialists, faculty, practitioners, and students during teleconsultation submissions (Fig. 3).

A rubric created by the faculty was utilized to evaluate teleconsultation submissions, ensuring both consistency and rigor in the assessment process. The rubric (Table 1) includes five essential criteria: clarity of the case, accuracy in diagnosis, therapeutic planning, punctuality, and the efficient use of telemedicine. Each criterion was assessed on a four-tier scale to offer structured feedback and recognize areas needing enhancement.

**Table 1.** Clinical Case Evaluation Rubric for Teleconsultation Cases

Criterion	Description	Weight (%)	Performance levels			
			Excellent (90–100)	Good (75–89)	Needs improvement (60–74)	Unsatisfactory (<60)
Case clarity	Completeness and organization of the case, including history, findings, and clinical context.	20%	Comprehensive and well-organized; all necessary details are present.	Clear and mostly complete; minor details missing.	Key details are missing or poorly organized.	Incomplete or disorganized; difficult to understand.
Diagnostic accuracy	Appropriateness and justification of the proposed diagnosis based on provided information.	25%	Accurate diagnosis with clear, evidence-based justification.	Accurate diagnosis with minor justification gaps.	Diagnosis is partially accurate or lacks sufficient evidence.	Diagnosis is incorrect or unsupported.
Therapeutic plan	Relevance and completeness of the proposed treatment, including adherence to guidelines.	25%	Evidence-based, complete, and appropriate plan tailored to the patient’s condition.	Appropriate plan with minor omissions or lack of detail.	Plan is incomplete or partially relevant.	Plan is inappropriate or lacks essential components.
Timeliness	Timely case submission and response.	15%	Case submitted and responded to promptly, within the defined time frame.	Slight delays that do not impact patient care.	Delays that may affect patient care.	Significant delays compromising patient care.
Use of telemedicine features	Effective utilization of platform tools, such as attachments, test requests, and messaging, to enhance case quality.	15%	All relevant features are used effectively to support the case.	Effectively used with minor gaps.	Features are underutilized, limiting case quality.	Features are not used or misused, compromising the case submission.

### Data collection and analysis

Data collection included surveys, response time analysis, and thematic sentiment analysis.^12^ A mixed-method approach was used to analyze the data collected.

#### Quantitative analysis

Descriptive statistics (mean, variance, standard deviation) assessed task completion times and user ratings. Multiple-choice questions on ease of use and perceived effectiveness, measured on a 5-point Likert scale, structured the evaluation of participant experiences. The questions included (1) Do you think that sending and receiving teleconsultations improves your ability to diagnose and manage gynecological cases? (2) How easy was it for you to use an asynchronous telemedicine platform to send and receive teleconsultations? (3) Clarity of the feedback provided by the faculty.

#### Qualitative analysis

The open-ended responses to the surveys were thematically analyzed using QuestionPro sentiment analysis tools to identify key trends and user feedback. Key trends were identified based on the participants’ answers to the following questions: (1) How has this experience influenced your understanding and skills in the management of gynecological cases? (2) Can you provide a concrete example of something you have learned or improved on?

#### Cross-validation

Five specialists independently reviewed diagnoses and case presentations during clinical validation using a faculty-designed rubric to ensure consistency in evaluating clarity, diagnostic precision, and additional comments.

#### Ethical considerations

Informed consent was obtained from all participants. Data anonymity was maintained by coding and secure storage. The study complied with local and international data protection standards with regular audits to ensure ethical compliance.^34^

## Results

### Functional testing results

Functional testing of the telemedicine system was performed between August 2022 and October 2023. This phase involved structured evaluations with 10 general practitioners and five specialty physicians from rural areas, focusing on task completion time, error rates, and system usability to assess the platform’s performance in simulated real-world conditions. The test scenarios were designed without real patient data to ensure a controlled environment for evaluating the functionality of the system. Data collection included quantitative metrics, such as task completion times and error rates, and qualitative insights gathered through user satisfaction surveys and feedback sessions (Table 2).

**Table 2.** Summary of Functionality Testing Results

Metrics	Mobile application (rural general practitioners)	Web platform (specialist doctors)
Task completion time	5 min (avg)	3 min (avg)
Error rate	2%	1%
User satisfaction (1–5, Likert scale)	4.5	4.7
Key issues identified	Offline data sync	Case review workflow

Qualitative feedback highlighted strengths like an intuitive interface, offline data recording, and improved rural medical care. Challenges included data synchronization issues, case filtering, and the need for more training materials, offering insights for further system refinement.

Improvements to the telemedicine system, guided by user feedback and testing, focused on functionality and usability. Updates included enhanced data synchronization, advanced filtering for case reviews, comprehensive training materials, improved interface navigation, and back-end optimizations to reduce latency and boost responsiveness, addressing user-identified issues.

### Clinical validation results

The clinical validation phase (October 2022–December 2023) assessed the telemedicine platform’s effectiveness through three teleconsultations in specialties like pediatrics and dermatology. Quantitative data showed 100% diagnostic precision, an average response time of 24 h, and positive outcomes in all cases, demonstrating the platform’s reliability in rural health care. Real clinical cases, including pediatric neurodevelopmental concerns and dermatological conditions such as herpes zoster, are summarized (Table 3), with specialists providing targeted recommendations. Qualitative feedback highlighted the platform’s role in enhancing patient care and supporting accurate diagnosis and effective management in underserved areas.

**Table 3.** Summary of Real Clinical Cases

Case type	Clinical case	Consultation	Specialist response	External specialist comments
Pediatric case	6-year-old girl with learning difficulties and suspected microcephaly.	Detailed history and physical examination provided.	Recommendations for neurodevelopmental assessment and educational support.	Suggested vision and hearing evaluations, thyroid function tests, and developmental assessments. Consideration of congenital infections.
Dermatology Case 1	36-year-old man with vesicular lesions on the back.	Symptoms suggestive of herpes zoster.	Diagnosis confirmed; acyclovir and analgesics prescribed.	Confirmed herpes zoster diagnosis and supported initial diagnosis by rural practitioner.
Dermatology Case 2	45-year-old woman with a chronic foot lesion.	Lesion described with suspicion of leprosy.	Differential diagnosis provided; recommended culture and biopsy.	Suggested chromoblastomycosis or cutaneous tuberculosis; long-term treatment or standard tuberculosis treatment recommended.

To further validate the teleconsultation findings, an independent validation process was conducted using a cross-validation method with five medical specialists: two dermatologists, one pediatrician, and two gynecologists. This process was facilitated through the QuestionPro tool, allowing each specialist to independently review and evaluate the accuracy of the diagnoses and clarity of the clinical case presentations.

#### Diagnostic accuracy

Overall, 80% of the specialists confirmed that the diagnoses provided were accurate according to current clinical guidelines and their professional judgment. A specialist suggested that pediatric cases should include an analysis of potential malnutrition as a contributing factor to learning difficulties.

#### Clarity of the case

Overall, 80% of the specialists found the clinical information in the cases to be clearly presented. Suggestions for improvement include better descriptions and high-resolution images for dermatological cases to enhance diagnostic accuracy.

#### Additional comments

Some specialists recommend adding more detailed diagnostic options and allowing continuous updates of clinical information as new data become available. Others emphasized the importance of including high-quality images and suggested the integration of teleconsultation capabilities for real-time examinations.

### Educational validation results

**FIG. 4.**
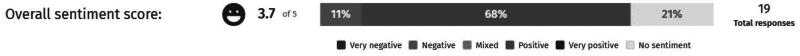
Impact of telemedicine on the learning of gynecology: sentiment analysis of participants.

The educational validation, conducted during the academic period from April to August, assessed the platform’s integration into medical education and its impact on diagnostic skills. Students completed 64 gynecological teleconsultations, evaluated by faculty based on delivery timelines and case quality, with results integrated into course grading. Teleconsultations demonstrated high diagnostic accuracy and a practical average specialist response time of 18 h. The validation results (Table 4) and a sentiment analysis highlight a positive perception of the platform’s effectiveness in enhancing clinical learning and gynecological skills, with an overall score of 3.7 out of 5 and 68% of responses classified as positive or very positive (Fig. 4).

**Table 4.** Educational Validation Results of the Telemedicine App

Metric	Value
Number of teleconsultations	64
Specialties	Gynecology
Average response time	18 h
Accurate diagnoses provided	100%
Follow-up improvement	Positive outcomes in all cases
Perceived effectiveness and usefulness of telemedicine for gynecology learning	Very useful: 57.14% Moderately useful: 33.33% Neutral: 9.52%
General sentiment and satisfaction about the telemedicine platform	Average sentiment: 3.8/5 Positive: 85% Negative: 5% Without answering: 10%

Diagnoses reviewed during the educational validation phase were classified using the ICD-10 system, highlighting a diverse range of clinical cases. In particular, 25% of the cases were related to absent, sparse, or rare menstruation (N91), 8% to candidiasis (B37), and 6% to other abnormal uterine or vaginal bleeding (N93). Endometriosis (N80) accounted for 5% of the cases, while the remaining 56% covered various other conditions. This diversity underscores the ability of the platform to simulate a wide range of gynecological scenarios, broaden the diagnostic exposure of students, and better prepare them for real-world clinical practice.^35^

Finally, the evaluation of 64 teleconsultations revealed that diagnostic precision achieved the highest proportion of excellent scores (28%), reflecting the students’ ability to correlate clinical findings with accurate diagnoses. However, therapeutic plans presented significant challenges, with 36% rated as unsatisfactory, highlighting the need for further training in treatment formulations. The use of telemedicine features was rated as good in 38% of cases, suggesting moderate proficiency in using the platform effectively. These findings underscore both the strengths and areas of improvement in the integration of teleconsultations into medical education.

## Discussion

This study demonstrated the feasibility and effectiveness of a WATP geared for rural health care and medical education in Ecuador. Unlike previous efforts that primarily evaluated telemedicine for clinical purposes, this research is notable for its integration of telemedicine training into the medical curriculum, equipping students with essential skills to handle real-world challenges.^7,8,12^

WATP differentiates itself from platforms like MedConsult, the application developed by Hanash et al.^36^ by incorporating offline capabilities and an educational feature that enhances the diagnostic competencies of medical students. Its iterative design, tailored to rural needs, combines health care delivery and education into a unique, scalable model that addresses the limitations of platforms focused solely on consultations.^37^

The clinical validation phase evaluated three teleconsultations (one pediatric and two dermatological) representing prevalent conditions in the Cayapas River region, where limited access to clean water and sanitation contributed to high rates of infectious and dermatological diseases.^38,39^ Cross-validation by five independent specialists confirmed a 100% diagnostic accuracy rate, highlighting the reliability of the platform. To strengthen these findings, future studies should include a wider range of clinical cases and assess the performance of the platform in diverse health care settings.

The results demonstrated the effectiveness of the platform in improving clinical preparedness and diagnostic skills, with gynecological cases such as absent or rare menstruation (ICD-10: N91) representing 25% of diagnoses and diagnostic precision receiving the highest scores.^40,41^ However, Faculty evaluations reported low performance in therapeutic planning, highlighting the need for enhanced training in treatment formulation and platform utilization to optimize its educational impact. Expanding its use to disciplines such as nursing and dentistry could further broaden its scope and foster interdisciplinary collaboration.^42,43^

Despite the promising outcomes, this study encountered several limitations. The limited sample size during clinical validation, which was affected by political instability and restricted access to the Cayapas region, constrains its generalizability.^44,45^ Correspondingly, the educational validation centered on a single gynecology course narrows the range of teleconsultation scenarios. Nevertheless, the project is undergoing a continuous evaluation phase until February 2026, with intentions to incorporate additional disciplines such as internal medicine, surgery, and pediatrics, and to extend its execution to other universities within Ecuador. As part of these efforts, an iOS version of the platform has been developed and will follow a phased validation process: first in the controlled environment of SIMUEES, then in hospitals, and eventually in rural areas. These initiatives aim to increase the breadth of clinical scenarios, participant diversity, and platform accessibility, thereby enhancing the study’s representativeness and impact.^46,47^

## Conclusions

This study assessed a WATP for functionality, clinical accuracy, and educational value. It proved effective with reliable performance in resource-limited areas, precise teleconsultation diagnoses, and improved diagnostic skills in medical students, highlighting its potential to reduce health care inequities and enhance medical education.

## Abbreviations Used

TMP: telemedicine platform
UI: user interface
UEES: Universidad Espíritu Santo
UML: unified modeling language
WATP: Web- and Android-based Telemedicine Platform
